# Strontium Ranelate Inhibits Osteoclastogenesis through NF-κB-Pathway-Dependent Autophagy

**DOI:** 10.3390/bioengineering10030365

**Published:** 2023-03-16

**Authors:** Dongle Wu, Xuan Sun, Yiwei Zhao, Yuanbo Liu, Ziqi Gan, Zhen Zhang, Xin Chen, Yang Cao

**Affiliations:** 1 Hospital of Stomatology, Guanghua School of Stomatology, Sun Yat-Sen University, Guangzhou 510055, China; 2 Guangdong Provincial Key Laboratory of Stomatology, Guangzhou 510080, China

**Keywords:** strontium ranelate, orthodontic tooth movement, osteoclast, NF-κB pathway, autophagy

## Abstract

Strontium ranelate (SR) is a pharmaceutical agent used for the prevention and treatment of osteoporosis and fragility fracture. However, little attention has been paid to the effect of SR on alveolar bone remodeling during orthodontic tooth movement and its underlying mechanism. Here, we investigated the influence of SR on orthodontic tooth movement and tooth resorption in Sprague–Dawley rats and the relationship between the nuclear factor–kappa B (NF-κB) pathway, autophagy, and osteoclastogenesis after the administration of SR in vitro and in vivo. In this study, it was found that SR reduced the expression of autophagy-related proteins at the pressure side of the first molars during orthodontic tooth movement. Similarly, the expression of these autophagy-related proteins and the size and number of autophagosomes were downregulated by SR in vitro. The results also showed that SR reduced the number of osteoclasts and suppressed orthodontic tooth movement and root resorption in rats, which could be partially restored using rapamycin, an autophagy inducer. Autophagy was attenuated after pre-osteoclasts were treated with Bay 11-7082, an NF-κB pathway inhibitor, while SR reduced the expression of the proteins central to the NF-κB pathway. Collectively, this study revealed that SR might suppress osteoclastogenesis through NF-κB-pathway-dependent autophagy, resulting in the inhibition of orthodontic tooth movement and root resorption in rats, which might offer a new insight into the treatment of malocclusion and bone metabolic diseases.

## 1. Introduction

Nowadays, an increasing number of people, especially female adults in high-income countries, such as Germany, are turning to orthodontic treatment for oral health and aesthetic appearance [1,2]. However, it has been found clinically that the orthodontic tooth movement of patients who have taken anti-osteoporosis drugs, such as bisphosphonate and strontium, seems to slow down compared to others.

Strontium ranelate (SR) is a potent pharmaceutical agent used for the prevention and treatment of osteoporosis and fragility fracture. It has a unique dual mechanism in bone tissue that promotes bone formation and inhibits bone resorption simultaneously [3]. Specifically, it can promote osteoblastogenesis and the mineralization of the bone matrix, while inhibiting the migration, accumulation, differentiation, and resorption ability of osteoclasts, so as to reduce the risk of fracture and increase bone strength [4,5]. In the field of stomatology, SR has been proven to improve dental implant osseointegration, attenuate root resorption during orthodontic tooth movement, and reduce alveolar bone resorption in rats with periodontitis [6,7,8]. Nevertheless, little attention has been paid to the effect of SR on orthodontic tooth movement, and its specific mechanism of action has not been fully elucidated, which needs to be further investigated.

Previous studies have shown that SR can inhibit the proliferation and differentiation of osteoclasts via the nuclear factor–kappa B (NF-κB) pathway [9,10]. This transcription factor family functions as a critical regulatory factor in inflammation, immune response, and bone metabolism [11,12,13]. In the classic NF-κB pathway, p65 and p50 are released from their trimer after the p-inhibitor of kappa B kinase β (p-IKKβ) phosphorylates the inhibitor of kappa B α (IκBα). Next, p65 and p50 transfer into the nucleus and activate the transcription of downstream genes, resulting in the enhancement of osteoblastogenesis and the inhibition of osteoclastogenesis [11]. Accumulating evidence indicates that SR suppresses tumor-necrosis-factor-α-induced NF-κB activation in pre-osteoblasts and nuclear translocation of NF-κB regulated by the receptor activator of nuclear factor-kappa B ligand (RANKL) in pre-osteoclasts, which promotes osteoblast differentiation and attenuates osteoclast differentiation eventually [10,14].

Meanwhile, the NF-κB pathway has been proven to play an important role in autophagy, and the strong link between autophagy and osteoclastogenesis is being increasingly appreciated [15]. Autophagy, a highly conserved catabolic process in eukaryotes, is responsible for phagocytosing and degrading damaged organelles or aged proteins via the lysosomal pathway. After cytoplasmic substances, such as macromolecules, organelles, and exogenous pathogens, are phagocytosed into autophagic bodies, they fuse with lysosomes to form autolysosomes that degrade and recycle the contents of autophagic bodies, providing energy and nutrition for the repair, survival, and maintenance of cells [16]. A number of studies have revealed that autophagy has different effects on the proliferation, differentiation, and activity of bone cells, including osteocytes, osteoblasts, and osteoclasts [17,18,19]. The dysfunction of autophagy may cause abnormality of bone homeostasis, thus resulting in various diseases associated with bone metabolism [20]. Furthermore, it has been demonstrated that the NF-κB pathway can directly enhance autophagy by inducing the expression of autophagy-related proteins, such as autophagy-related 5 homolog (ATG5), microtubule-associated protein light chain 3 (LC3), and Beclin1. These proteins are reported to be highly involved in osteoclast activity, including the formation of a ruffled border, the transport of lysosomes, and the release of protease in vitro and in vivo [21,22,23,24]. Nevertheless, the role of the NF-κB pathway and autophagy in the mechanism of osteoclastogenesis inhibited by SR has not been studied yet.

This study aimed to determine the effect of SR on the efficiency of orthodontic tooth movement so as to provide a prediction of the therapy time of orthodontic patients who have taken SR. In addition, the pharmaceutical mechanism of SR was further elucidated in this study, which might provide evidence for the treatment of bone metabolic diseases.

## 2. Materials and Methods

### 2.1. Ethical Approval

This study was conducted with approval from the Institutional Animal Care and Use Committee (IACUC), Sun Yat-sen University (no. SYSU-IACUC-2021-000015).

### 2.2. Osteoclast Culture

After bone marrow cells were harvested from the femur and tibia of 4-week-old Sprague–Dawley rats, they were lysed with red blood cell lysis (Cwbio, Taizhou, China) on ice, centrifuged, resuspended in high-glucose Dulbecco’s modified Eagle medium (Gibco, Grand Island, NE, USA) containing 10% fetal bovine serum (Gibco), and cultured in a 5% CO_2_ incubator at 37 °C for 2 days. The rat bone-marrow-derived macrophages in the culture medium were collected, centrifuged, and resuspended in complete medium containing 30 ng/mL of macrophage-colony-stimulating factor (MCSF; PeproTech, Cranbury, NJ, USA) and then cultured in an incubator at 37 °C for 3 days to induce pre-osteoclasts. The pre-osteoclasts were divided into three groups and resuspended in a medium containing 30 ng/mL of MCSF and 50 ng/mL of RANKL (R & D Systems, Minneapolis, MN, USA). At the same time, the SR group also received 2 mM SR (Maclin, Shanghai, China), while the SR + rapamycin (RAPA; TargetMol, Boston, MA, USA) group received 2 mM SR and 0.1 nM RAPA, an autophagic inducer. After culture in an incubator at 37 °C for 5 days, osteoclasts were collected for tartrate-resistant acid phosphatase (TRAP) staining, monodansylcadaverine staining, Western blot analysis, and transmission electron microscope observation.

### 2.3. In Vitro TRAP Staining

TRAP is a specific enzyme produced in osteoclasts, which can be used to identify these multinucleated giant cells. After the culture medium was removed, the cells were fixed with 4% paraformaldehyde (Servicebio, Wuhan, China) for 30 min, cultured with TRAP dye solution (Sigma-Aldrich, Darmstadt, Germany) at 37 °C for 30 min in the dark, and counted under a light microscope (Axio; Zeiss, Oberkochen, Germany).

### 2.4. Western Blot

After the cells were lysed with radio immunoprecipitation assay (RIPA) lysis buffer (CST, Boston, MA, USA) on ice for 30 min, the concentration of protein was calculated with the bicinchoninic acid protein assay kit (Beyotime, Shanghai, China). RIPA lysis buffer and sodium dodecyl sulfate–polyacrylamide (Beyotime) were used to dilute the proteins to obtain an equal concentration. Next, they were denatured in boiling water for 10 min; loaded in 12% MOPS electrophoretic gel (GenScript, Piscataway, NJ, USA) in the same amount; transferred onto a polyvinylidene fluoride membrane (Millipore, Burlington, MA, USA); blocked with 5% bovine serum albumin (Servicebio); incubated with the first antibody at 4 °C overnight, including tumor-necrosis-factor-receptor-associated factor 6 (TRAF6; 1:500; Servicebio), c-Fos (1:1000; Servicebio), matrix metalloproteinases-9 (MMP-9; 1:1000; CST), matrix metalloproteinase-14 (MMP-14; 1:500; Zen, Shanghai, China), cathepsin K (CTSK; 1:500; Servicebio), Beclin1 (1:500; Boster, Wuhan, China), LC3 (1:1000; CST), lysosomal-associated membrane protein 2 (LAMP2; 1:500; Zen), ATG5 (1:500; Zen), p62 (1:500; Boster), p-IKKα/β (1:500; Affinity, Shanghai, China), IκBα (1:500; Zen), and p65 (1:500; Zen); incubated with horseradish peroxidase (HRP)-labeled goat anti-rabbit IgG (1:500; Servicebio) at 25 °C for an hour; and finally visualized with a chemiluminescent substrate (Millipore) using a chemiluminescence imaging system (ChemiDoc; Bio-Rad, Hercules, CA, USA).

### 2.5. Monodansylcadaverine Staining

Monodansylcadaverine is a fluorescent stain usually used to detect the formation of autophagosomes. After the induction of osteoclastogenesis, these cells were cultured with 10% monodansylcadaverine (Leagene, Beijing, China) at 37 °C for 50 min in the dark and then observed under a laser confocal microscope (LSM980; Zeiss).

### 2.6. Transmission Electron Microscopy

After the culture medium was removed, the osteoclasts were separated with trypsin (Gibco) for 10 min, centrifuged for 2 min, and immersed in 2.5% glutaraldehyde (Servicebio) for 30 min. Next, they were embedded with 1% agarose solution (Servicebio); fixed with 1% osmic acid (TPI, Reading, PA, USA) for 2 h in the dark; dehydrated with 30%, 50%, 70%, 80%, 95%, and 100% ethanol (Servicebio) and 100% acetone (Servicebio); embedded with acetone and 812 (SPI, Shanghai, China) at 37 °C overnight; polymerized at 60 °C for 48 h; sectioned into 60-nm-thick slides using an ultra-microtome (UC7, Wetzlar, Germany, Leica); stained with 2% uranium acetate (Servicebio) and 2.6% lead citrate (Servicebio) for 8 min each; and observed under a transmission electron microscope (HT7800; Hitachi, Tokyo, Japan).

### 2.7. Animal Maintenance

Forty-five 10-week-old male Sprague–Dawley rats (Sun Yat-sen University, Guangzhou, China) were used in this experiment. These rats weighed between 200 g and 220 g and were provided with a semi-liquid diet all day under specific pathogen-free conditions. They were randomly divided into 3 groups of 15 rats each: control group, SR group, and SR + RAPA group.

### 2.8. Orthodontic Tooth Movement Model

After the induction of general anesthesia with 3% pentobarbital sodium (30 mg/kg; CVRI, Shanghai, China), surgeries were performed on the maxillary left bones of the rats in all 3 groups. An orthodontic ligature wire made of stainless steel (Xinya, Hangzhou, China) was inserted through the undercut between the first and the second maxillary left molar. The wire was ligated with an orthodontic nitinol spring (Xinya), and another ligature wire was fastened on the other side of the spring. After the spring was extended with a dynamometer (Tiantian, Hangzhou, China) until the force reached 0.3 N, the wire on the other side was ligated with the incisors. Chemically cured resin was used to further fasten the ligature of the wire and the labial surfaces of the incisors after the surfaces were acid-etched (Heraeus Kulzer GmbH, Germany), washed, and dried and a primer applied to them (3M Unitek, Saint Paul, MN, USA) [25].

### 2.9. Drug Treatment in Rats

Drug administration began 3 weeks before the establishment of the orthodontic tooth movement models and lasted until the rats were sacrificed in all groups. The rats in the SR group were administered SR dissolved in 1.5 mL of physiological saline at a dosage of 900 mg/kg through gastric tubes every day. The rats in the SR + RAPA group received 900 mg/kg of SR and 300 μg/kg of RAPA, and the rats in the control group received only an equal amount of physiological saline using the same method.

### 2.10. Sample Collection and Treatment

Five rats from each group were euthanized using general anesthesia on days 3, 7, and 14. After the maxillary left alveolar bones of the rats were removed, they were scanned with micro-CT (voltage: 85 kV, current: 200 μA, resolution ratio: 10 μm; SkyScan 1276; Bruker, Karlsruhe, Germany) for analysis of orthodontic tooth movement, tooth resorption, and trabecular structure. Next, the bones were fixed with 4% paraformaldehyde (Servicebio) for 24 h; decalcified with ethylene diamine tetraacetic acid (EDTA; Servicebio) for several weeks; dehydrated with 75%, 85%, 90%, 95%, and 100% ethanol (Servicebio), alcohol benzene (Servicebio), and xylene (Servicebio) in an automatic tissue processor (Donatello; DIAPATH, Shanghai, China); and embedded with molten paraffin (Servicebio) in an embedding machine (JB-P5; Junjie, Wuhan, China). After cooling and trimmed, these wax blocks were sectioned into 4-μm-thick slides along the sagittal direction with a pathology slicer (RM2016; Leica), which were then used for hematoxylin-eosin (HE) staining, TRAP staining, immunohistochemistry, and immunofluorescence.

### 2.11. Analysis of Data Obtained with Micro-CT

Micro-CT data were analyzed using CTAn 1.20 (Bruker), a high-resolution microscopic CT quantitative analysis software [26,27]. Orthodontic tooth movement was calculated by measuring the distance between the protruding point of the first molar distal wall and the second molar mesial wall. The volume of root resorption at the pressure side of the first molars was calculated with CTAn 1.20, and 3D images of resorption were reconstructed with CTVox 3.3 (Bruker), a 3D CT reconstruction software. The basic micro-architectural parameters of the bone trabecula at the pressure side of the first molars were used to analyze the changes in the trabecular structure, including the bone volume/total volume, trabecular number, trabecular thickness, and trabecular separation.

### 2.12. Hematoxylin-Eosin Staining

Dewaxed sections were incubated with hematoxylin (Servicebio) and eosin (Servicebio); dehydrated with xylene, anhydrous alcohol, and 75% ethanol; and sealed with neutral gum (Servicebio). The cytoplasm and nucleus of all the cells turned to red and blue, respectively. The slices were scanned (Aperio AT2, Leica), and osteoclasts and tooth resorption lacuna were observed at the pressure side of the upper-left first molars.

### 2.13. In Vivo Tartrate-Resistant Acid Phosphatase Staining

After the dewaxed sections were incubated with distilled water at 37 °C for 2 h and TRAP incubation solution at 37 °C for 20 min, they were stained with hematoxylin; dehydrated with xylene, anhydrous alcohol, and 75% ethanol; and finally sealed with neutral gum. The cytoplasm and nucleus of TRAP-positive cells turned wine red and light blue, respectively. The slices were scanned (Aperio AT2, Leica), and TRAP-positive multinucleated cells were observed and counted at the pressure side of the upper-left first molars.

### 2.14. Immunohistochemistry

The dewaxed sections were immersed in EDTA antigen retrieval buffer (Servicebio), heated in a microwave (P70D20TL-P4, Glanze, Hangzhou, China) at sub-boiling temperature for 15 min for antigen retrieval, incubated with 3% hydrogen peroxide (Servicebio) at 25 °C for 25 min to block endogenous peroxidase, and mixed with 3% bovine serum albumin (Servicebio) at 25 °C for 30 min in the dark to be blocked by serum. After that, the slices were incubated with a primary antibody at 4 °C overnight, including RANK (1:100; Zen), osteoprotegerin (OPG; 1:200; Servicebio), TRAF6 (1:100; Servicebio), nuclear factor of activated T cells 2 (NFATc2; 1:500; Servicebio), c-Fos (1:400; Servicebio), MMP-14 (1:100; Zen), CTSK (1:1000; Servicebio), p-IKK α/β (1:100; Affinity), IκBα (1:100; Zen), p65 (1:200; Zen), Beclin1 (1:200; Boster), LC3 (1:500; CST), LAMP2 (1:100; Zen), ATG5 (1:50; Zen), and p62 (1:200; Boster). Next, they were incubated with HRP-labeled goat anti-rabbit IgG (1:200; Servicebio) at 25 °C for 50 min; mixed with diaminobenzidine (DAB; Servicebio) solution for coloration; counterstained with hematoxylin; dehydrated with xylene, anhydrous alcohol, and 75% ethanol; and finally sealed with neutral gum. They were scanned (Aperio AT2; Leica), and the positive expression appeared brownish yellow. The mean optical density (MOD) was measured using Image-Pro Plus 6.0 (Media Cybernetics, Rockville, MD, USA) to represent the signal intensity of the positive expression.

### 2.15. Immunofluorescence

All procedures were conducted in the dark according to a previous study [24]. For single labeling of immunofluorescence, after antigen retrieval, endogenous peroxidase blocking, and serum blocking, as described before, the dewaxed sections were incubated with a primary antibody at 4 °C overnight, including Beclin1 (1:100; Boster), LC3 (1:100; CST), LAMP2 (1:100; Zen), ATG5 (1:50; Zen), and p62 (1:300; Boster), and then incubated with Cy3-labeled goat anti-rabbit IgG (1:300; Servicebio) at 25 °C for 50 min. For triple labeling of immunofluorescence, after antigen retrieval and serum blocking, as described before, the dewaxed sections were incubated with p-IKKα/β (1:1000; Affinity) at 4 °C overnight, HRP-labeled goat anti-rabbit IgG (1:300; Servicebio) at 25 °C for 50 min, and Cy3-TSA solution (1:300; Servicebio) at 25 °C for 10 min; immersed in EDTA antigen retrieval buffer (Servicebio); and heated in a microwave (P70D20TL-P4; Glanze) at sub-boiling temperature for 15 min. Next, the slices were incubated with IκBα (1:3000; Zen) at 4 °C overnight, HRP-labeled goat anti-rabbit IgG at 25 °C for 50 min, and 488-TSA solution (1:500; Servicebio) at 25 °C for 50 min and heated in the microwave, as described before. After that, they were incubated with p65 (1:200; Zen) at 4 °C overnight and Cy5-labeled goat anti-mouse IgG (1:400; Servicebio) at 25 °C for 50 min. Finally, the slices for single labeling and triple labeling were counterstained with DAPI solution (Servicebio) at 25 °C for 10 min, mixed with spontaneous fluorescence quenching reagent (Servicebio) at 25 °C for 5 min, sealed with anti-fade mounting medium (Servicebio), and scanned (3Dhistech, Pannoramic, Budapest, Hungary) to obtain high-resolution images. The mean fluorescence intensity (MFI) of the positive expression was measured using Image-Pro Plus 6.0 (Media Cybernetics).

### 2.16. Statistics

All experiments were repeated three times, and quantitative results were presented as the mean ± standard deviation (SD). All data were obtained by two examiners who were blinded to the groups, and analyzed with one-way analysis of variance (ANOVA) followed by the Bonferroni test in SPSS 23.0 (IBM, Chicago, IL, USA). *p* < 0.05 was considered statistically significant.

## 3. Results

### 3.1. Strontium Ranelate Inhibited Osteoclastogenesis through Autophagy

**Figure 1:**
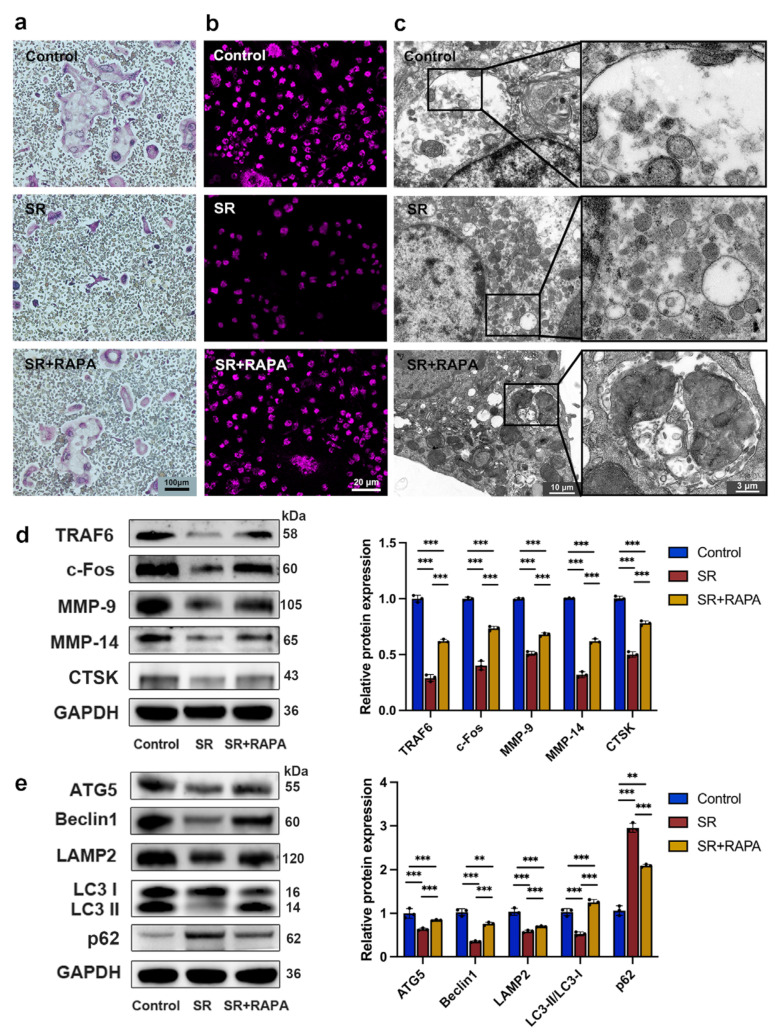
Strontium ranelate suppressed osteoclast differentiation through autophagy in vitro. (**a**) TRAP staining images after pre-osteoclasts received different treatments for 5 days. (**b**) Monodansylcadaverine staining images after pre-osteoclasts received different treatments for 5 days. (**c**) Representative images of autophagosomes captured with a transmission electron microscope after pre-osteoclasts received different treatments for 5 days. Western blot assay and relative protein expression of the osteoclast markers TRAF6, c-Fos, MMP-9, MMP-14, and CTSK (**d**) and the autophagic proteins Beclin1, ATG5, LAMP2, LC3-I, LC3-II, and p62 (**e**) after pre-osteoclasts received different treatments for 5 days. (n = 3, mean ± SD, ** *p* < 0.01, *** *p* < 0.001).

After pre-osteoclasts were treated with SR for 5 days, TRAP staining showed that the nucleus number and the number and size of multinucleated giant cells decreased in comparison with the control group (Figure 1a and Figure S1 in Supplementary Materials). As shown in Figure 1d, the expression levels of TRAF6, c-Fos, MMP-9, MMP-14, and CTSK, the osteoclast markers, which were detected with Western blot analysis, reduced in the SR group. Monodansylcadaverine staining (Figure 1b and Figure S2 in Supplementary Materials) showed that the MFI of the autophagosome, including the autophagic body with a two-layer membrane and the autolysosome with a one-layer membrane, decreased after the addition of SR, and transmission electron microscope observation (Figure 1c and Figure S3 in Supplementary Materials) exhibited that the size and number of autophagosomes reduced significantly in comparison with the control group. It was found that the expression levels of Beclin1, ATG5, LAMP2, and LC3-II/LC3-I in the SR group were lower than those in the control group, while the expression level of p62, a regulator and substrate of autophagy, increased in the SR group compared with the control group, according to the analysis of Western blot (Figure 1e). Furthermore, monodansylcadaverine staining (Figure 1b and Figure S2 in Supplementary Materials) and transmission electron microscope observation (Figure 1c and Figure S3 in Supplementary Materials) showed that RAPA, a macrolide antibiotic that activates the process of autophagy, restored in part the MFI and the size and number of autophagosomes attenuated by SR. The expression levels of osteoclast markers (Figure 1d), including TRAF6, c-Fos, MMP-9, MMP-14, and CTSK, and autophagy-related proteins (Figure 1e), such as Beclin1, ATG5, LAMP2, LC3-II/LC3-I, and p62, were partially restored after the pre-osteoclasts were treated with RAPA in comparison with the SR group. These results suggested that RAPA might enhance the proliferation, differentiation, and function of osteoclasts suppressed by SR, indicating that SR might inhibit osteoclastogenesis through autophagy.

### 3.2. Strontium Ranelate Inhibited Autophagy through the NF-κB Pathway

**Figure 2:**
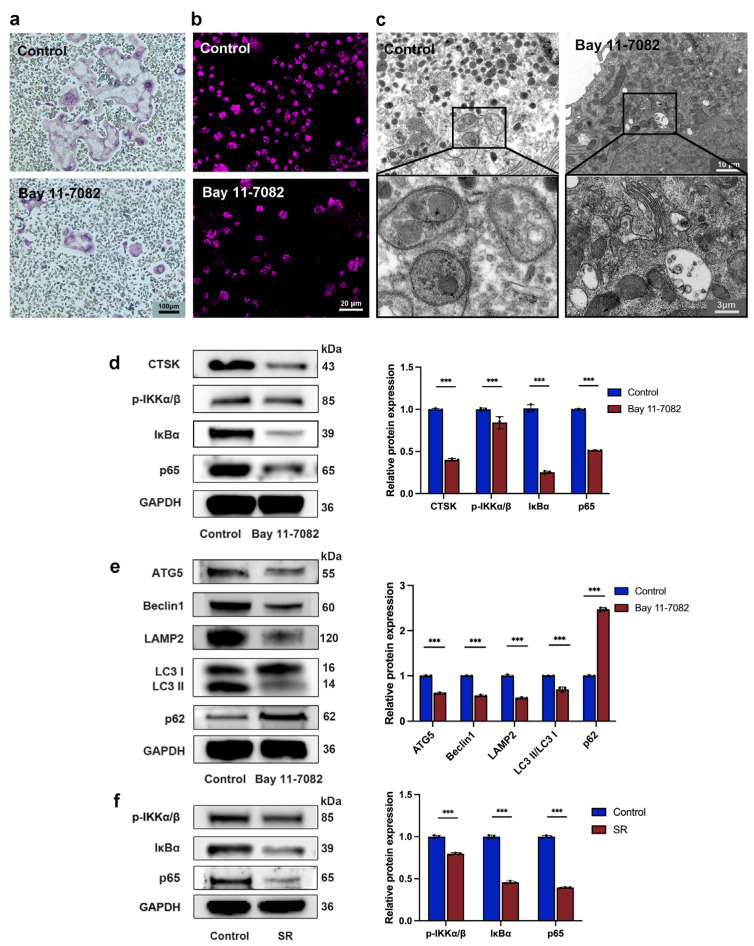
Strontium ranelate inhibited osteoclast differentiation through NF-κB-pathway-dependent autophagy in vitro. (**a**) TRAP staining images after pre-osteoclasts received different treatments for 5 days. (**b**) Monodansylcadaverine staining images after pre-osteoclasts received different treatments for 5 days. (**c**) Representative images of autophagosomes captured with a transmission electron microscope after pre-osteoclasts received different treatments for 5 days. Western blot assay and relative protein expression of the osteoclast marker CTSK, the proteins central to the NF-κB pathway (p-IKKα/β, IκBα, and p65) (**d**), and the autophagic proteins Beclin-1, ATG5, LAMP2, LC3-I, LC3-II, and p62 (**e**) after pre-osteoclasts were treated with Bay 11-7082 for 5 days. (**f**) Western blot assay and relative protein expression of the proteins central to the NF-κB pathway after pre-osteoclasts were treated with SR for 5 days. (n = 3, mean ± SD, *** *p* < 0.001).

There is much evidence demonstrating that the NF-κB pathway plays an essential role in osteoclastogenesis suppressed by SR and has relevance to the process of autophagy, so we next investigated whether SR inhibits autophagy via this pathway. TRAP staining showed that Bay 11-7082, a suppressor of the NF-κB pathway, attenuated the nucleus number and the number and size of osteoclasts (Figure 2a and Figure S4 in Supplementary Materials). The size and number of autophagosomes decreased due to the inhibitor, according to the analysis of monodansylcadaverine staining (Figure 2b and Figure S5 in Supplementary Materials) and transmission electron microscope observation (Figure 2c and Figure S6 in Supplementary Materials). This inhibitor also reduced the expression levels of the osteoclast marker CTSK and the proteins central to the NF-κB pathway, including p-IKKα/β, IκBα, and p65 (Figure 2d). As shown in Figure 2e, Bay 11-7082 downregulated the expression levels of autophagy-related proteins, including Beclin1, ATG5, LAMP2, and LC3-II/LC3-I, and enhanced the expression level of p62. Furthermore, after osteoclast precursors were treated with SR for 5 days, Western blot analysis showed that the expression levels of p-IKKα/β, IκBα, and p65 significantly reduced compared to the control group (Figure 2f). These results revealed that SR might attenuate autophagy through the NF-κB pathway, which further decreases the proliferation and differentiation of osteoclasts.

### 3.3. Strontium Ranelate Reduced Orthodontic Tooth Movement and Root Resorption in Rats through Autophagy

**Figure 3:**
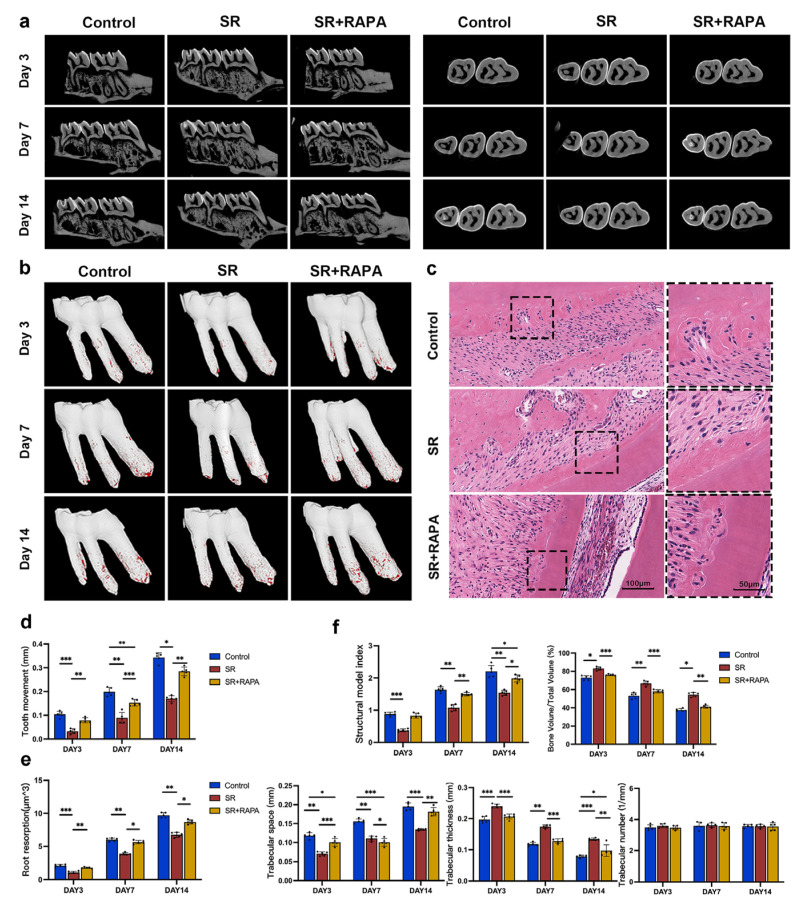
Strontium ranelate reduced orthodontic tooth movement and root resorption in rats. Three-dimensionally reconstructed micro-CT images of orthodontic tooth movement (**a**) and root resorption at the pressure side of the first molars (**b**) on days 3, 7, and 14 in different groups after orthodontic movement devices were established in rats. (**c**) HE staining images of root resorption at the pressure side of the first molars on day 7 in different groups. Quantitative analyses of orthodontic tooth movement (**d**), root resorption (**e**), and structure model index, thickness, bone volume/total volume, and trabecular space of the trabecular bone (**f**) at the pressure side of the first molars on days 3, 7, and 14 in different groups after orthodontic movement devices were established. (n = 5, mean ± SD, * *p* < 0.05, ** *p* < 0.01, *** *p* < 0.001).

Five rats from each group were sacrificed on days 3, 7, and 14, and their maxillary left alveolar bones were removed and then scanned with micro-CT. As shown in Figure 3a, the first molars moved faster from day 0 to day 7 than from day 7 to day 14 in all groups. The administration of SR significantly slowed down the velocity of tooth movement compared to the control group (Figure 3a,d). It was found that the thickness and bone volume/total volume of trabecular bone at the pressure side of the first molars increased, while the structure model index and trabecular space reduced after the administration of SR, according to trabecular structural analysis, indicating that SR enhances the micro-architecture of trabecular bone (Figure 3f). HE staining (Figure 3c) and micro-CT (Figure 3b,e) showed that the size and volume of root resorption at the pressure side of the first molars were significantly attenuated in the SR group. In addition, RAPA partially restored the effect of SR, including the velocity of tooth movement (Figure 3a,d), the micro-architecture of trabecular bone (Figure 3f), and the extent of root resorption (Figure 3b,c,e).

### 3.4. Strontium Ranelate Might Inhibit Osteoclastogenesis through NF-κB-Mediated Autophagy in Sprague–Dawley Rats

**Figure 4:**
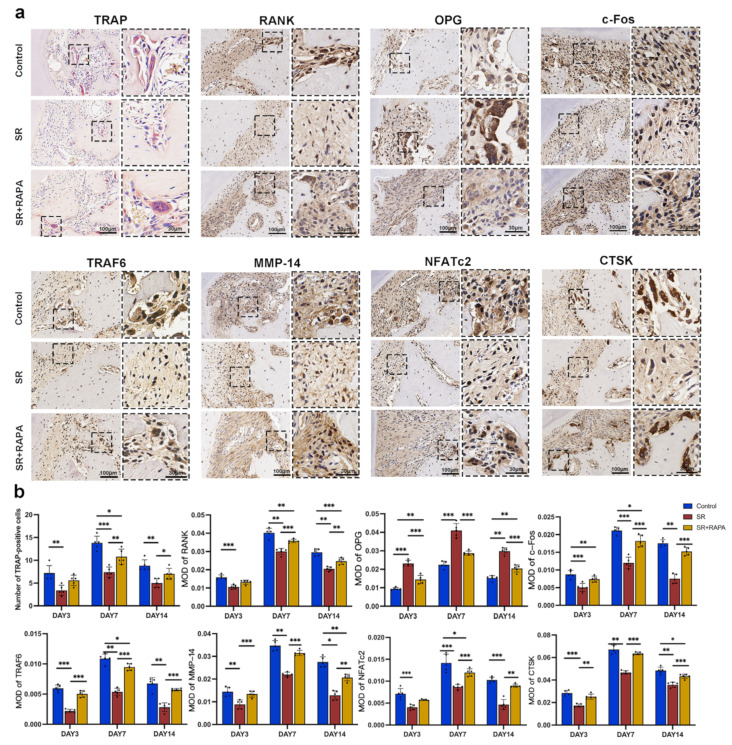
Strontium ranelate inhibited osteoclast differentiation and the expression of osteoclast markers in rats. (**a**) TRAP staining images of osteoclasts and immunohistochemical staining images of RANK, OPG, c-Fos, TRAF6, MMP-14, NFATc2, and CTSK at the pressure side of the first molars on day 7 in different groups. (**b**) Number of osteoclasts and the mean optical density (MOD) of RANK, OPG, c-Fos, TRAF6, MMP-14, NFATc2, and CTSK at the pressure side of the first molars on days 3, 7, and 14 in different groups. (n = 5, mean ± SD, * *p* < 0.05, ** *p* < 0.01, *** *p* < 0.001).

TRAP-staining showed that the number of osteoclasts decreased after the administration of SR at the pressure side of the first molars. Immunohistochemistry analysis showed that osteoclast markers, including RANK, TRAF6, NFATc2, c-Fos, MMP-14, and CTSK, had lower positive expression levels, while OPG, a negative regulator of osteoclast differentiation, had a higher positive expression level after the rats were treated with SR in comparison with the control group (Figure 4a,b).

**Figure 5:**
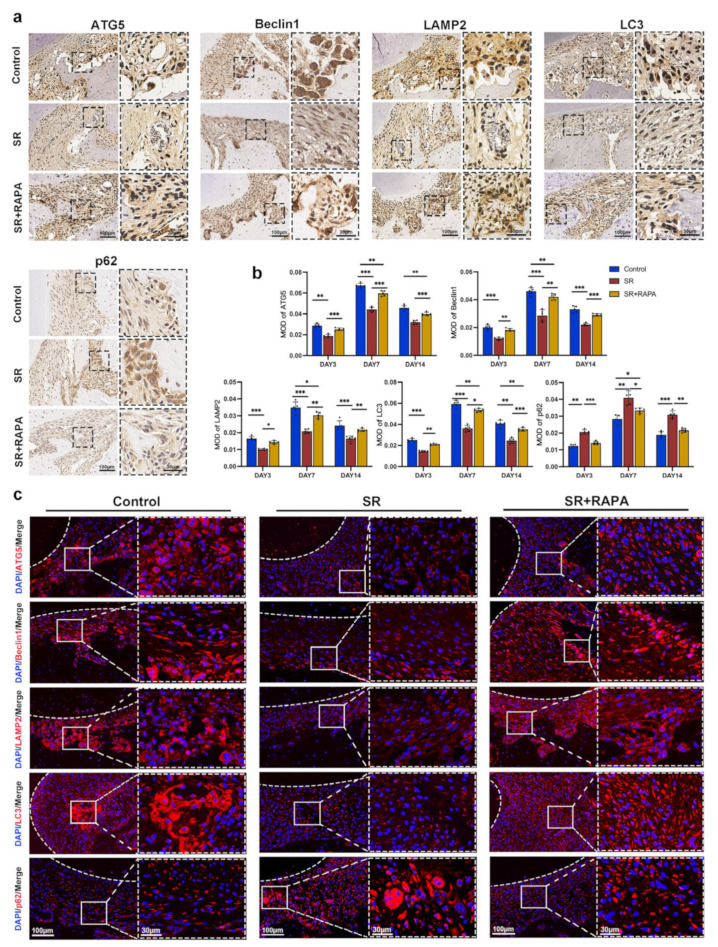
Strontium ranelate regulated the expression of autophagic proteins in rats. (**a**) Immunohistochemical staining images of ATG5, Beclin1, LAMP2, LC3, and p62 at the pressure side of the first molars on day 7 in different groups. (**b**) The mean optical density (MOD) of ATG5, Beclin1, LAMP2, LC3, and p62 at the pressure side of the first molars on days 3, 7, and 14 in different groups. (n = 5, mean ± SD, * *p* < 0.05, ** *p* < 0.01, *** *p* < 0.001). (**c**) Immunofluorescence staining images of ATG5, Beclin1, LAMP2, LC3, and p62 at the pressure side of the first molars on day 7 in different groups. The white dashed lines indicate the margin of the tooth root.

SR also downregulated the MOD and MFI of autophagy-related proteins, including Beclin1, ATG5, LAMP2, and LC3, while it enhanced the MOD and MFI of p62 at the pressure side of the first molars, according to the analysis of immunohistochemistry (Figure 5a,b) and immunofluorescence (Figure 5c and Figure S7 in Supplementary Materials). In addition, RAPA partially restored the number of osteoclasts (Figure 4a,b); the MOD of the osteoclast markers (Figure 4a,b), including RANK, TRAF6, NFATc2, c-Fos, MMP-14, and CTSK; and the MOD (Figure 5a,b) and MFI (Figure 5c and Figure S7 in Supplementary Materials) of the autophagy-related proteins, such as Beclin1, ATG5, LAMP2, LC3, and p62, at the pressure side of the first molars.

**Figure 6:**
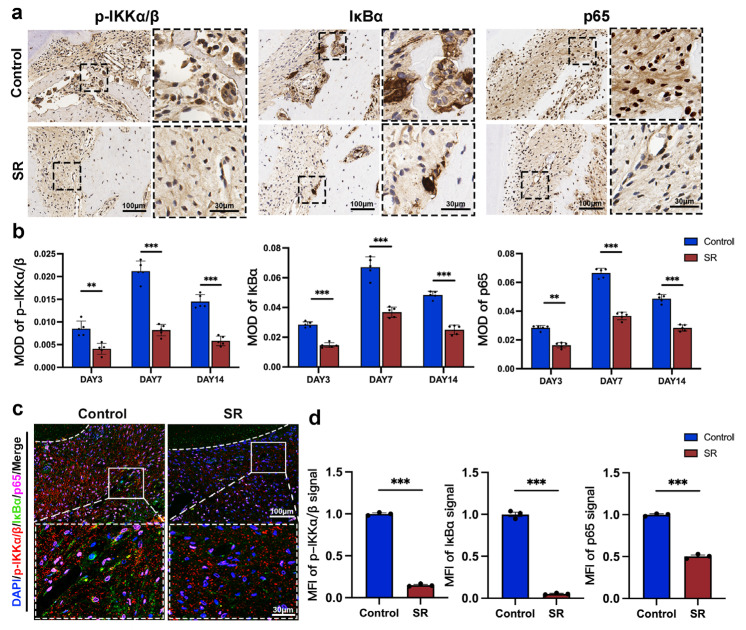
Strontium ranelate suppressed the expression of proteins central to the NF-κB pathway in rats. (**a**) Immunohistochemical staining images of p-IKKα/β, IκBα, and p65 at the pressure side of the first molars on day 7 in different groups. The mean optical density (MOD) (**b**) and mean fluorescence intensity (MFI) (**d**) of p-IKKα/β, IκBα, and p65 at the pressure side of the first molars on days 3, 7, and 14 in different groups. (n = 5, mean ± SD, ** *p* < 0.01, *** *p* < 0.001). (**c**) Immunofluorescence staining images of p-IKKα/β, IκBα, and p65 at the pressure side of the first molars on day 7 in different groups. The white dashed lines indicate the margin of the tooth root.

**Figure 7:**
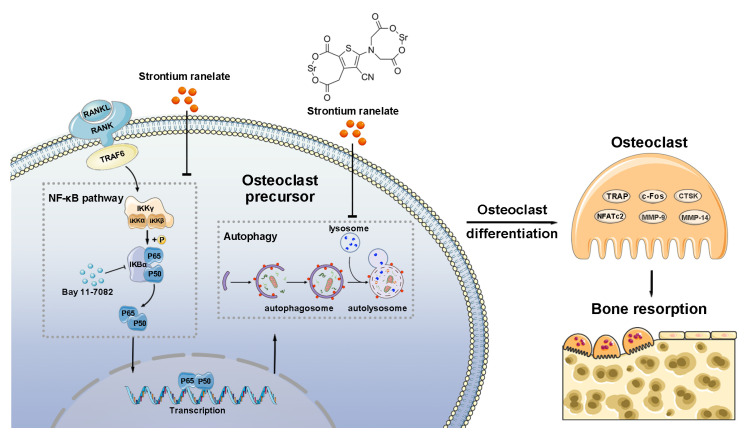
Schematic illustration of the mechanism of how SR suppresses osteoclastogenesis by inhibiting autophagy through the NF-κB pathway.

The MOD (Figure 6a,b) and MFI (Figure 6c,d) of the proteins central to the NF-κB pathway, including p-IKKα/β, IκBα, and p65, reduced after the administration of SR, according to the analysis of immunohistochemistry and immunofluorescence. Immunofluorescence assay also showed that the transport of p65 from the cytoplasm to the cell nucleus was downregulated in the SR group (Figure 6c). These findings indicated that SR might suppress osteoclast differentiation at the pressure side of the first molars in rats through the activation of NF-κB-pathway-dependent autophagy, leading to the inhibition of orthodontic tooth movement and root resorption (Figure 7).

## 4. Discussion

Under many physiological and pathological conditions, autophagy is initiated to protect cells for repair, survival, and maintenance [28]. In the process of orthodontic tooth movement, the periodontal ligament at the pressure side and the blood vessels inside it are compressed. These changes result in narrowed periodontal space and reduced blood flow, which puts the pre-osteoclasts at the pressure side under an anoxic state [29]. Under hypoxia stress, osteoclast precursors may initiate autophagy to protect them for survival and prevent them from undergoing apoptosis [30]. Autophagy is initiated through a compound containing ATG13 and then nucleated by a Beclin1 compound to form an autophagosome [31]. During the process of elongation, LC3-I in the cytoplasm is converted to LC3-II by ATG5 and other autophagic proteins [32]. Previous studies have shown that the expression levels of ATG5, Beclin1, and LC3 increase, while the expression level of p62, the substrate of autophagy, reduces at the pressure side of the first molars in adult male mice during orthodontic tooth movement. These results indicate that autophagy is indeed activated during the process [30,33]. In this study, an orthodontic tooth movement model was established in Sprague–Dawley rats. It was found that the administration of SR downregulated the expression levels of ATG5, Beclin1, LAMP2 (a specific marker in the lysosome membrane), and LC3-II/LC3-I and upregulated the expression level of p62 in vitro and in vivo. SR also attenuated the number and size of autolysosomes in osteoclasts in vitro, according to the analysis of monodansylcadaverine staining and transmission electron microscope observation, which revealed that the autophagy of osteoclasts at the pressure side of orthodontic tooth movement is inhibited under the influence of SR [34].

Furthermore, the differentiation and function of osteoclasts have been reported to be positively correlated with their autophagic activity. A number of studies have demonstrated that the deficiency of these autophagy-related proteins could inhibit osteoclastogenesis [19,22,35]. It was also found that sphingosine-1-phosphate (S1P), a lipid signaling molecular that promotes cell growth and inhibits apoptosis, regulates not only the mammalian target of RAPA (mTOR) pathway but also the migration of osteoclasts. These studies have suggested that S1P is a bond between autophagy and osteoclast accumulation [36]. There is evidence revealing that ATG5, LC3, and other autophagic proteins have a significant influence on the resorption ability of osteoclasts, including the formation of a ruffled border, the fusion of lysosomes and the plasma membrane, and the secretion of proteases, such as members of the MMP family and CTSK. These results indicate that autophagy could enhance the bone resorption ability of osteoclasts [21,22,37]. In this study, the administration of SR inhibited the differentiation and function of osteoclasts at the pressure side of the first molars. It was found that the nucleus number and the number and size of the red multinucleated giant cells significantly decreased in vitro and in vivo, according to the analysis of TRAP staining, a specific marker enzyme of osteoclasts. Various osteoclast markers reduced in the SR group, including RANK, TRAF6, NFATc2, c-Fos, and some proteases related to bone resorption, such as MMP-9, MMP-14, and CTSK. These proteases could degrade extracellular matrix protein, including collagen and elastin, break intercellular connections, and promote cell development and migration [15,35,38,39,40] In addition, orthodontic tooth movement at the pressure side of the first molars was inhibited in the SR group, according to the analysis of 3D reconstruction with micro-CT. The inhibition of tooth movement might result from the fact that SR significantly enhances the micro-architecture of trabecular bone by attenuating osteoclast differentiation. Although SR slowed down the process of orthodontic treatment, it decreased the extent of root resorption during the tooth movement, which suggests that topical SR might be a potent pharmaceutical agent for the prevention and treatment of root resorption. However, these effects of SR could be partially reversed by RAPA, an autophagy activator that inhibits mTOR complex 1. These results indicated that SR might suppress proliferation, differentiation and function of osteoclast through autophagy, leading to the inhibition of orthodontic tooth movement and root resorption.

However, several lines of evidence suggest that autophagy plays a negative role in osteoclastogenesis [41,42]. Likewise, it has also been found that autophagy has a dual effect on the differentiation and activity of osteocytes and osteoblasts during bone remodeling [18,43,44,45] As osteocytes and osteoblasts can influence the development, function, and survival of osteoclasts through the RANK/RANKL/OPG pathway, it becomes difficult when assessing the role of autophagy in osteoclastogenesis, resulting in the contradictory conclusions in previous studies [46,47,48]. It was found that autophagy regulates osteoclastogenesis in a dose-dependent manner. The result showed that the proliferation, differentiation, and function of osteoclasts are enhanced at a low autophagic level, while they are attenuated with an increasing autophagic level [49]. Therefore, we suspect that different levels of autophagy might have contradictory effects on osteocytes, osteoblasts, and osteoclasts under different states of disease or external stimuli in vivo and in vitro. In this study, the results showed that autophagy is positively correlated with the proliferation and differentiation of osteoclasts at the pressure side of the first molars during orthodontic tooth movement, indicating that the effect of autophagy might lead to osteoclastogenesis under this circumstance. Nevertheless, the exact mechanism of autophagy in osteoclasts is still unclear and needs to be further elucidated.

It has been demonstrated that the NF-κB pathway plays an essential part in the development, function, and survival of osteoclasts inhibited by SR and is related to the process of autophagy, which suggests that the NF-κB pathway might be a critical link between SR and autophagy [9,10,15]. In this study, SR decreased the expression levels of the proteins central to the NF-κB pathway, including p-IKKα/β, IκBα, and p65, in vivo and in vitro. It might function through the RANK/RANKL/OPG axis, a classical pathway of osteoclastogenesis, that antagonizes the NF-κB pathway, according to previous studies [15]. Bay 11-7082, an inhibitor of the NF-κB pathway, downregulated the autophagy-related proteins except p62 and decreased the size and number of autophagosomes, based on the analysis of monodansylcadaverine staining and transmission electron microscope observation. Similarly, there is evidence that IKK/NF-κB directly enhances autophagy by inducing the expression of related proteins such as ATG5, LC3, and Beclin1 [23,50]. These results demonstrate that SR attenuates osteoclastogenesis by inhibiting autophagy through the NF-κB pathway.

There are several limitations to this study. First, we concentrated on only the effect of SR on osteoclasts instead of osteoblasts, which also play an essential part in bone remodeling. Second, SR has some severe side effects, such as thromboembolism, but most of them can be avoided if physicians strictly follow the indications of SR [51]. It was reported that physicians strictly control the indications of SR, such as severe osteoporosis with a high absolute fracture risk, significantly reducing the occurrence of these side effects [52]. Last, the exact mechanisms of how the NF-κB pathway regulates autophagy and how autophagy affects osteoclastogenesis are still unclear and need to be further elucidated.

While an increasing number of people are turning to orthodontic treatment, including some osteoporotic patients who have taken SR, little attention has been paid to the effect of SR on orthodontic tooth movement and its underlying mechanism. In this study, it was found that SR could suppress osteoclastogenesis by inhibiting autophagy through the NF-κB pathway, leading to the inhibition of orthodontic tooth movement and root resorption in Sprague–Dawley rats. Therefore, orthodontists should be aware that patients who are taking SR will have a longer course of treatment and need to be informed of their condition in advance. Topical administration of SR might be a more promising and practical mode of administration in orthodontic treatment to prevent orthodontic side effects, such as anchor loss and tooth resorption, and thus avoid the use of inconvenient extraoral anchorage and invasive surgery. However, considering that SR is difficult to penetrate through alveolar bone if injected into the mucosa directly, it is important to find a more appropriate method to allow SR to affect specific regions during orthodontic treatment.

## 5. Conclusions

This study revealed that SR might attenuate osteoclastogenesis through NF-κB-pathway-dependent autophagy, resulting in the inhibition of orthodontic tooth movement and root resorption in rats, which might offer a new insight into the treatment of malocclusion and bone metabolic diseases.

## Data Availability

Data are available within the article.

## Supplementary Materials

The following supporting information can be downloaded at https://www.mdpi.com/article/10.3390/bioengineering10030365/s1: Figure S1: The number of osteoclasts, the number of nuclei per osteoclast, and the mean size of osteoclasts after pre-osteoclasts were treated for 5 days in the control group, the strontium ranelate (SR) group, and the SR + rapamycin (RAPA) group; Figure S2: The mean fluorescence intensity (MFI) of autophagosomes after pre-osteoclasts received different treatments for 5 days; Figure S3: The number and mean size of autophagosomes after pre-osteoclasts received different treatments for 5 days; Figure S4: The number of osteoclasts, the number of nuclei per osteoclast, and the mean size of osteoclasts after pre-osteoclasts received different treatments for 5 days; Figure S5: The mean fluorescence intensity (MFI) of autophagosomes after pre-osteoclasts received different treatments for 5 days; Figure S6: The number and mean size of autophagosomes after pre-osteoclasts received different treatments for 5 days; Figure S7: The mean fluorescence intensity (MFI) of ATG5, Beclin1, LAMP2, LC3, and p62 at the pressure side of the first molars on day 7 in different groups.
