# Supplementary material for: Strontium Ranelate Inhibits Osteoclastogenesis through NF-κB-Pathway-Dependent Autophagy
Source: Bioengineering (Basel). 2023 Mar 16;10(3):365. doi: 10.3390/bioengineering10030365 (PMC10045081; doi:10.3390/bioengineering10030365)
Supplement: Supplementary file 1 [file bioengineering-10-00365-s001.zip › bioengineering-2184649-supplementary.pdf]

bioengineering

Supplementary Materials

Strontium Ranelate Inhibits Osteoclastogenesis Through NF-κB-Pathway-Dependent Autophagy

Dongle Wu [1,2,†], Xuan Sun [1,2,†], Yiwei Zhao [1,2,†], Yuanbo Liu [1,2], Ziqi Gan [1,2], Zhen Zhang [1,2], Xin Chen [1,2] and Yang Cao [1,2,*]

1 Hospital of Stomatology, Guanghua School of Stomatology, Sun Yat-sen University, Guangzhou 510055, China
2 Guangdong Provincial Key Laboratory of Stomatology, Guangzhou 510080, China
* Correspondence: caoyang@mail.sysu.edu.cn
† These authors contributed equally to this work.

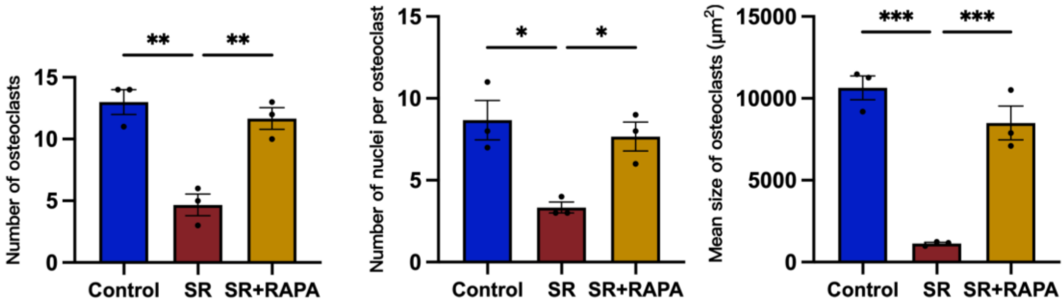

Figure S1. The number of osteoclasts, the number of nuclei per osteoclast, and the mean size of osteoclasts after pre-osteoclasts were treated for 5 days in the control group, the strontium ranelate (SR) group, and the SR+rapamycin (RAPA) group . (n = 3, mean ± SD, *P <0.05, **P <0.01, ***P <0.001).

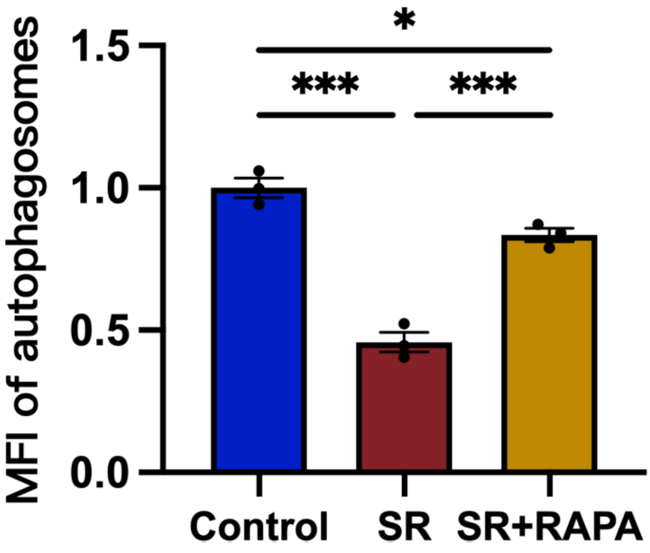

Figure S2. The mean fluorescence intensity (MFI) of autophagosomes after pre-osteoclasts received different treatments for 5 days. (n = 3, mean ± SD, *P <0.05, ***P <0.001).

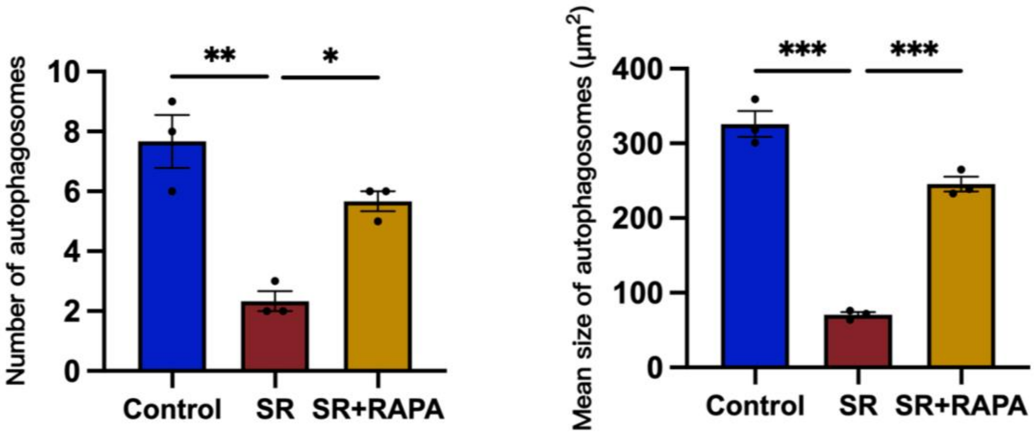

Figure S3. The number and mean size of autophagosomes after pre-osteoclasts received different treatments for 5 days. (n = 3, mean ± SD, *P <0.05, **P <0.01, ***P <0.001).

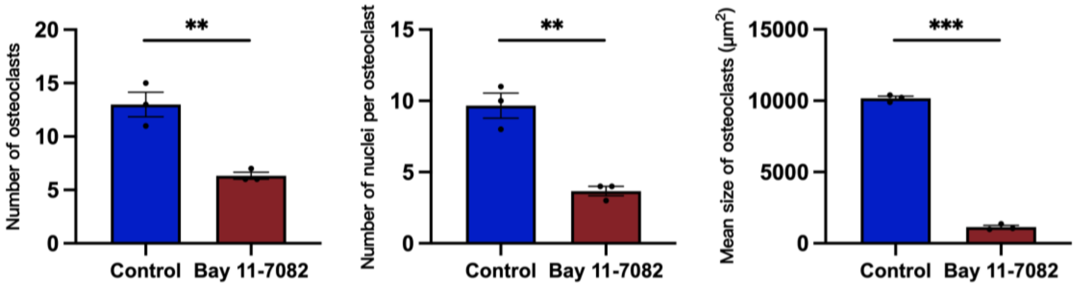

Figure S4. The number of osteoclasts, the number of nuclei per osteoclast, and the mean size of osteoclasts after pre-osteoclasts received different treatments for 5 days. (n = 3, mean ± SD, **P <0.01, ***P <0.001).

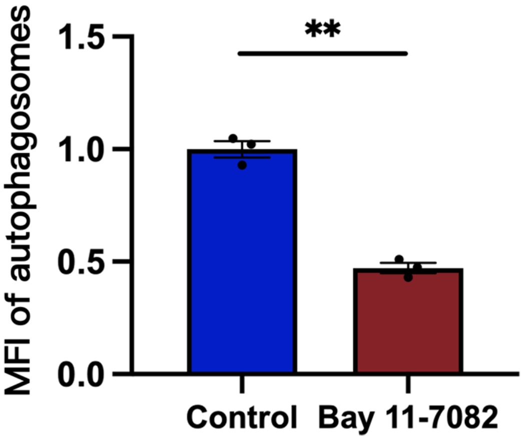

Figure S5. The mean fluorescence intensity (MFI) of autophagosomes after pre-osteoclasts received different treatments for 5 days. (n = 3, mean ± SD, *P <0.05, ***P <0.001).

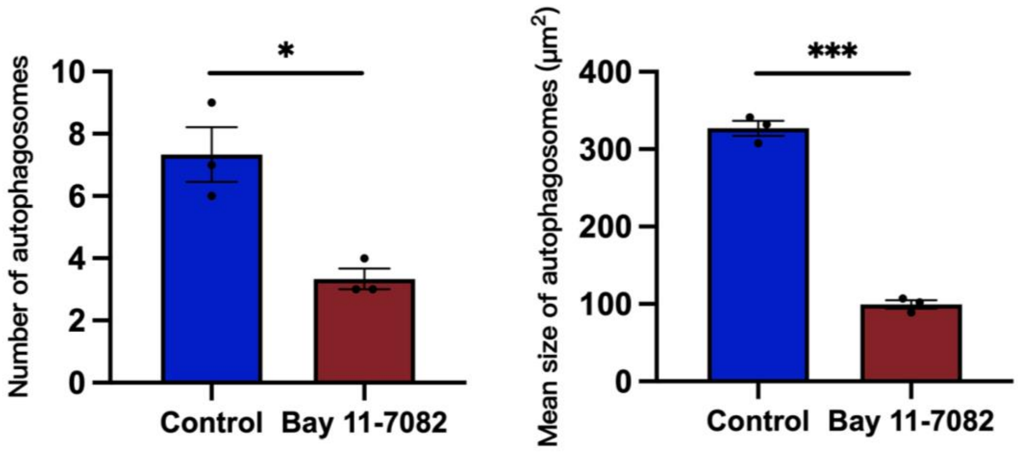

Figure S6. The number and mean size of autophagosomes after pre-osteoclasts received different treatments for 5 days. (n = 3, mean ± SD, *P <0.05, ***P <0.001).

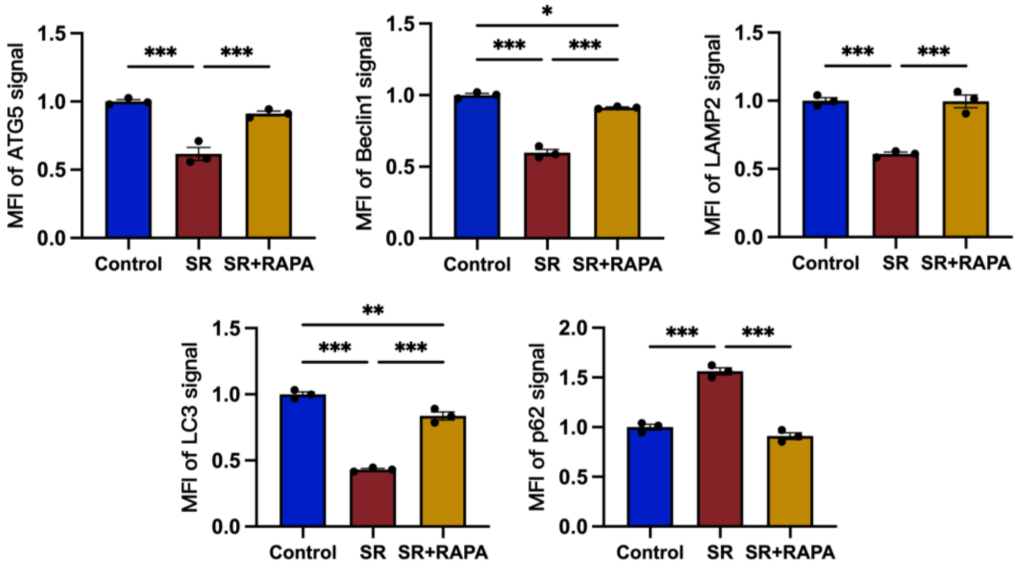

Figure S7. The mean fluorescence intensity (MFI) of ATG5, Beclin1, LAMP2, LC3, and p62 at the pressure side of the first molars on day 7 in different groups. (n = 5, mean ± SD, *P <0.05, **P <0.01, ***P <0.001).
